# Supplementary material for: Diversity Analysis of Tick-Borne Viruses from Hedgehogs and Hares in Qingdao, China
Source: Microbiol Spectr. 2023 Apr 19;11(3):e05340-22. doi: 10.1128/spectrum.05340-22 (PMC10269667; doi:10.1128/spectrum.05340-22)

Supplementary Table 1. List of tick pools and results of NGS

Pool.no	Host	Spiece(n)	No.of reads	No.of RNA reads	Total number of sequences	Total length of sequences (bp)	Shortest sequence length (bp)	Longest sequence length (bp)	N50	Overall alignment rate
H01	Hedgehog	H.flava (75)	89399894	661	28564	12855779	200	8551	440(10549 sequences)	57.50%
H02	Hedgehog	H.flava(80)	110542414	3929	3519	1666975	201	12112	441(1188 sequences)	66.51%
H03	Hedgehog	R.sanguineus (62)	77487458	193749	9733	4425248	200	22344	430(3533 sequences)	56.95%
H04	Hedgehog	D.sinicus(67)	70579492	1615	3956	1908458	200	9418	463(1316 sequences)	76.70%
H05	Hedgehog	H.longicornis(77)	86788540	609712	23348	11420046	200	12040	466(7823 sequences)	79.88%
H06	Hedgehog	H.campanulata(59)	96138282	1498	11563	5439204	200	9174	453(4012 sequences)	84.81%
R01	Rabbit	D.sinicus(60)	93150344	9548	2459	1157437	206	10187	442(813 sequences)	65.51%
R02	Rabbit	D.sinicus(60)	70064262	15194	3311	1582501	200	10186	456(1076 sequences)	75.68%
R03	Rabbit	D.sinicus(60)	81956484	21996	2963	1331109	200	10029	423(988 sequences)	86.21%
R04	Rabbit	D,sinicus(62)	77131206	15252	2279	994638	200	10193	428(755 sequences)	87.40%

Supplementary Table 2. List of tick pools and results of NGS

Virus	Primer	Sequence	Tm/℃	Length/bp
CPTV3	CPTV3-F	GCTGACGCGAGACGCTCATTT	62.3	21
	CPTV3-R	TCACAGACGTGTTTTCCGAATCG	60.7	23
	CPTV3-p	5'-FAM-CTCATTGACCCTTACTCGCTGCCTTTAGCT-BHQ1-3'	66.9	30
HBTV1	HBTV1-F	TAGAGTGACATTTGATAAAATTGAAAA	53.6	27
	HBTV1-R	ACTCCAAAATATTTTTTACAAGCC	54	24
	HBTV1-p	5'-FAM-TAAGACTCGTATGTTTTCGATGAGCCCTA-BHQ1-3'	62.8	29
HflFV	HflFV-F	GAGATTAATGATTTGGTTAATCAGC	56	25
	HflFV-R	AATCATCCCCATAGGTTATAACTC	57.4	24
	HflFV-p	5'-FAM-ATGCGTTATTGTTGGAAGAAAATAACACA-BHQ1-3'	58.5	29
HNTV	HNTV-F	GGAAGAAGGTAAGAAGAGCC	58.1	20
	HNTV-R	GCCTACCGTAAGCACTTTTG	58.1	20
	HNTV-p	5'-FAM-CTCTGCATCTTCTGTCTCCTCTGCACACA-BHQ1-3'	67	29
HPTV1	HPTV1-F	ACAGCTCAGTAACTTCCCCT	58.1	20
	HPTV1-R	AGTAAAACAAATGTTGGTCATTAGAGG	58.1	27

	HPTV1-p	5'-FAM-TTCAAGCATGCCAAGTGTCTGCACAGCA-BHQ1-3'	65.6	28
QDTIFV	QDTIFV-F	TTTAATTATAAGTGTTAGTGATAAAGC	53.6	27
	QDTIFV-R	GTAATATCACTCAAATCTCGTG	54.9	22
	QDTIFV-p	5'-FAM-AGATCGTTTTAATTTTTCTTCTATTCAAT-BHQ1-3'	54.3	29
QDTPV	QDTPV-F	TACTTAAAAAGATGTTGTGGGCATGC	59.5	26
	QDTPV-R	CAATGTGGCCATGGACTCC	59.9	19
	QDTPV-p	5'-FAM-ACCCTTCTTAGGAGGCTCTATGGGCAGGA-BHQ1-3'	68.4	29
QDTUV	QDTUV-F	ATTTCTCTGACCCAGAAGAGTC	58.6	22
	QDTUV-R	CTTCTGTCACCTCAAACCGAT	58.4	21
	QDTUV-p	5'-FAM-AGAGAGCCGTCCAACAAACCTTCCTAAGAA-BHQ1-3'	65.5	30
OKTV	OKTV-F	CAACATGACTCGTGCAAT	54	18
	OKTV-R	TTCCAGAGAGTGAGTTCTGA	53.7	20
	OKTV-p	5'-FAM-CATTGAAGTCTCTGAAGAACCAGT-BHQ1-3'	58.9	24

pool	NO.	Name	Length（bp）	Identity（%）	Query cover	Reference	Genebank
		**Supplementary Table 3 Nucleotide sequences used in this study**					
H01	virus1	Qingdao tick iflavirus strain H01	9195	73.83%	12%	Haemaphysalis flava iflavirus	LC483655.1
	virus2	Qingdao tick uukuvirus strain H01 L segment	6471	77.96%	95%	Toyo virus L	LC618931.1
		Qingdao tick uukuvirus strain H01 M segment	3053	72.79%	NA	Toyo virus M	LC618932.1
		Qingdao tick uukuvirus strain H01 S segment	1784	45.79%	NA	Toyo virus S	LC618933.1
	virus3	Okutama tick virus strain QDH01 L segment	6524	97.81%	100%	Okutama tick virus L	LC483653.1
		Okutama tick virus strain QDH01 S segment	1854	97.80%	100%	Okutama tick virus S	LC483654.1
H02	virus1	Qingdao tick iflavirus strain H02	9225	73.83%	12%	Haemaphysalis flava iflavirus	LC483655.1
	virus2	Huangpi Tick Virus 1 strain QDH02 L segment	11896	98.32%	100%	Huangpi Tick Virus 1 L	MW721865.1
		Huangpi Tick Virus 1 strain QDH02 M segment	4391	98.79%	99%	Huangpi Tick Virus 1 M	MW721866.1
		Huangpi Tick Virus 1 strain QDH02S segment	1915	99.01%	100%	Huangpi Tick Virus 1 S	MW721868.1
	virus3	Okutama tick virus strain QDH02 L segment	6561	98.13%	99%	Okutama tick virus L	LC483653.1
		Okutama tick virus strain QDH02 S segment	1854	98.25%	100%	Okutama tick virus S	LC483654.1
	virus4	Hubei tick virus strain QDH02	9117	87.43%	99%	Hubei tick virus 1	KX883731.1
	virus5	Henan tick virusstrain QDH02 L segment	12112	98.33%	100.00%	Henan tick virus	MZ244224.1
		Henan tick virusstrain QDH02 M segment	4806	94.53%	100.00%	Henan tick virus	MZ244226.1
		Henan tick virusstrain QDH02 S segment	2136	92.66%	100.00%	Henan tick virus	MZ244225.1
	virus6	k141_155	2556	69.82%	40%	Ixodes scapularis associated virus 2	KM048319.1
	virus7	k141_2304	2534	72.28%	85%	Ixodes scapularis associated virus 2	KM048319.1
H03	virus1	Changping Tick Virus 3 strain QDH03	10003	97.68%	93%	Changping Tick Virus 3	KM817595.1
	virus2	Okutama tick virus strain QDH03 L segment	6567	98.16%	99%	Okutama tick virus L	LC483653.1
		Okutama tick virus strain QDH03 S segment	1908	97.69%	100%	Okutama tick virus S	LC483654.1
	virus3	Hubei tick virus strain QDH03	9416	87.30%	99%	Hubei tick virus 1	KX883731.1
H04	virus1	Haemaphysalis flava iflavirus strain QDH04	9296	93.08%	97%	Haemaphysalis flava iflavirus	LC483655.1
	virus2	Hubei tick virus strain QDH04	9397	87.27%	99%	Hubei tick virus 1	KX883731.1
	virus3	Qingdao tick iflavirus strain H04	9165	76.42%	26%	Haemaphysalis flava iflavirus	LC483655.1
	virus4	Okutama tick virus strain QDH04 L segment	6961	97.85%	94%	Okutama tick virus L	LC483653.1
		Okutama tick virus strain QDH04 S segment	1759	97.61%	99%	Okutama tick virus S	LC483654.1
	virus5	Qingdao tick uukuvirus strain H04 L segment	6479	77.88%	95%	Toyo virus L	LC618931.1
		Qingdao tick uukuvirus strain H04 M segment	3053	72.73%	NA	Toyo virus M	LC618932.1
		Qingdao tick uukuvirus strain H04 S segment	1798	46.09%	NA	Toyo virus S	LC618933.1
		k141_4166	2577	70.08%	37%	Ixodes scapularis associated virus 2	KM048319.1
H05	virus1	Qingdao tick iflavirus strain H05	9190	73.83%	12%	Haemaphysalis flava iflavirus	LC483655.1
	virus2	Qingdao tick uukuvirus strain H05 L segment	6618	78.29%	74%	Toyo virus L	LC618931.1
		Qingdao tick uukuvirus strain H05 M segment	3053	72.48%	NA	Toyo virus M	LC618932.1
	virus3	Okutama tick virus strain QDH05 L segment	6634	98.81%	99%	Okutama tick virus L	LC483653.1
		Okutama tick virus strain QDH05 S segment	1957	97.75%	99%	Okutama tick virus S	LC483654.1
	virus4	Dabie bandavirus strain QDH05 L segment	6373	99.83%	99%	evere fever with thrombocytopenia syndrome virus	NC_043450.1
		Dabie bandavirus strain QDH05 M segment	3287	99.73%	100%	evere fever with thrombocytopenia syndrome virus	NC_043451.1
		Dabie bandavirus strain QDH05 S segment	1763	99.83%	97%	evere fever with thrombocytopenia syndrome virus	NC_043452.1
	virus5	k141_4166	2577	70.08%	37%	Ixodes scapularis associated virus 2	KM048319.1
	virus6	k141_4120	2587	69.92%	40%	Ixodes scapularis associated virus 2	KM048319.1
H06	virus1	Qingdao tick iflavirus strain H06	9174	73.83%	12%	Haemaphysalis flava iflavirus	LC483655.1
	virus2	Qingdao tick uukuvirus strain H06 L segment	6542	77.84%	94%	Toyo virus L	LC618931.1
		Qingdao tick uukuvirus strain H06 M segment	3053	72.70%	NA	Toyo virus M	LC618932.1
	virus3	Okutama tick virus strain QDH06 L segment	6765	98.10%	96%	Okutama tick virus L	LC483653.1
		Okutama tick virus strain QDH06 S segment	1854	97.80%	100%	Okutama tick virus S	LC483654.1
	virus4	k141_4106	2592	69.73%	40%	Ixodes scapularis associated virus 2	KM048319.1
	virus5	k141_1530	2600	78.28%	99%	Xinjiang tick associated virus 1	MH688544.1
R01	virus1	Qingdao tick phlebovirus strain R01 L segment	6412	98.91%	99%	Mukawa virus	NC_043510.1
		Qingdao tick phlebovirus strain R01 M segment	3330	98.59%	99%	Mukawa virus	NC_043509.1
		Qingdao tick phlebovirus strain R01 S segment	2165	99.03%	85%	Mukawa virus	NC_043511.1
	virus2	Hubei tick virus strain QDR01	8399	87.54%	99%	Hubei tick virus 1	KX883731.1
	virus3	Changping Tick Virus 3 strain QDR01	10045	97.85%	93%	Changping Tick Virus 3	KM817595.1
	virus4	Okutama tick virus strain QDR01 L segment	6675	98.07%	97%	Okutama tick virus L	LC483653.1
		Okutama tick virus strain QDR01 S segment	1847	97.74%	100%	Okutama tick virus S	LC483654.1
R02	virus1	Qingdao tick iflavirus strain R02	9169	76.30%	26%	Haemaphysalis flava iflavirus	LC483655.1
	virus2	Haemaphysalis flava iflavirus strain QDR02	9289	93.12%	98%	Haemaphysalis flava iflavirus	LC483655.1
	virus3	Changping Tick Virus 3 strain QDR02	10045	97.78%	93%	Changping Tick Virus 3	KM817595.1
	virus4	Okutama tick virus strain QDR02 L segment	6462	98.07%	100%	Okutama tick virus L	LC483653.1
		Okutama tick virus strain QDR02 S segment	1472	97.57%	96%	Okutama tick virus S	LC483653.1
	virus5	k141_3023	2606	78.34%	100%	Xinjiang tick associated virus 1	MH688544.1
	virus6	k141_5546	2586	69.95%	37%	Ixodes scapularis associated virus 2	KM048319.1
R03	virus1	Hubei tick virus strain QDR03	9463	87.10%	100%	Hubei tick virus 1	KX883731.1
	virus2	Changping Tick Virus 3 strain QDR03	10029	97.84%	93%	Changping Tick Virus 3	KM817595.1
R04	virus1	Hubei tick virus strain QDR04	8381	87.51%	99%	Hubei tick virus 1	KX883731.1
	virus2	Changping Tick Virus 3 strain QDR04	10052	97.76%	93%	Changping Tick Virus 3	KM817595.1
	virus3	Okutama tick virus strain QDR04 L segment	7044	97.92%	92%	Okutama tick virus L	LC483653.1
		Okutama tick virus strain QDR04 S segment	1857	97.74%	100%	Okutama tick virus S	LC483654.1

Supplementary Table 4 Amino acid sequences used in this study

RdRP			
**Family**	**Genus**	**Virus**	**Accession NO.**
iFlaviridae	*iflavirus*	Kakugo virus	BAD06930.1
iFlaviridae	*iflavirus*	Deformed wing virus	AFL65634.1
iFlaviridae	*iflavirus*	Varroa destructor virus	AAP51418.2
iFlaviridae	*iflavirus*	Bundaberg bee virus 6	AWK77862.1
iFlaviridae	*iflavirus*	Darwin bee virus 3	AWK77848.1
iFlaviridae	*iflavirus*	Formica exsecta virus 2	AHB62422.1
iFlaviridae	*iflavirus*	Hubei picorna-like virus 26	APG77410.1
iFlaviridae	*iflavirus*	Helicoverpa armigera iflavirus	APW84897.1
iFlaviridae	*iflavirus*	Heliconius erato iflavirus	AHW98099.1
iFlaviridae	*iflavirus*	Lymantria dispar iflavirus 1	AIF75200.1
iFlaviridae	*iflavirus*	Antheraea pernyi iflavirus	AH187751.1
iFlaviridae	*iflavirus*	Hubei odonate virus 4	APG78008.1
iFlaviridae	*iflavirus*	Moku virus	AOT85373.1
iFlaviridae	*iflavirus*	Hubeintorna-like virus 28	APG77971.1
iFlaviridae	*iflavirus*	Euscelidius variegatus virus 1	APD68841.1
iFlaviridae	*iflavirus*	Graminella nigrifrons virus 1	AJT58559.1
iFlaviridae	*iflavirus*	Pityohyphantes rubrofasciatus iflavirus	AQX17788.1
iFlaviridae	*iflavirus*	Brevicoryne brassicae virus	ABP57198.1
iFlaviridae	*iflavirus*	Nilaparvata lugens honeydew virus 1	BAN19725.1
iFlaviridae	*iflavirus*	Laodelphax striatellus picorna-like virus 2	AlX94679.1
iFlaviridae	*iflavirus*	Laodelphax striatella honeydewvirus 1	AHK05791.1
iFlaviridae	*iflavirus*	Shahe heteroptera virus 2	APG77409.1
iFlaviridae	*iflavirus*	Dinocampus coccinellae paralysis virus	AIM39350.1
iFlaviridae	*iflavirus*	Lampyris noctiluca iflavirus 2	QBP37020.1
iFlaviridae	*iflavirus*	Hubei picona-like virus 31	APG77963.1
iFlaviridae	*iflavirus*	Varroa destructor virus 2	APB88805.1
iFlaviridae	*iflavirus*	Nephila clavipes virus 1	AVK59473.1
iFlaviridae	*iflavirus*	Wuhan spider virus 2	APG77438.1
iFlaviridae	*iflavirus*	Tetranychus truncatus	AYV88988.1
iFlaviridae	*iflavirus*	Hubei tick virus 1	APG77503.1
iFlaviridae	*iflavirus*	Hubei tick virus 2	APG77502.1
iFlaviridae	*iflavirus*	lxodes scapularis iflavirus	BBD75427.1
iFlaviridae	*iflavirus*	Bole hyalomma asiaticum virus 1	APG77949.1
iFlaviridae	*iflavirus*	Hubei tick virus 3	APG77500.1
iFlaviridae	*iflavirus*	Haemaphysalis flava iflavirus	BBK20270.1
iFlaviridae	*iflavirus*	Hubei arthropod virus 1	APG77447.1
iFlaviridae	*iflavirus*	Lygus lineolaris virus 1	AEL30247.1
iFlaviridae	*iflavirus*	Sacbrood virus	AAD20260.1
iFlaviridae	*iflavirus*	Hubei picorna-like virus 38	APG77424.1
iFlaviridae	*iflavirus*	Infectious flacherie virus	BAA25371.1
iFlaviridae	*iflavirus*	Spodoptera exigua iflavirus 1	AET36829.1
iFlaviridae	*iflavirus*	Perina nuda virus	AAL06289.1
iFlaviridae	*iflavirus*	Spodoptera exigua iflavirus 2	AFQ98017.1
Phenuiviridae	*Rubodvirus*	Apple rubbery wood virus 1	AWC67511
Phenuiviridae	*Rubodvirus*	Apple rubbery wood virus 2	AWC67514
Phenuiviridae	*Entovirus*	Entoleuca phenui like virus 1	AVD68666
Phenuiviridae	*Lentinuvirus*	entinula edodes negative-strand RNA virus	BBI93118
Phenuiviridae	*Laulavirus*	Laurel Lake virus	YP_009667028
Phenuiviridae	*Coguvirus*	Citrus concave gum associated virus	YP_009422199
Phenuiviridae	*Mobuvirus*	Mothra bunyavirus	YP_009666266
Phenuiviridae	*Goukovirus*	Gouleako virus	YP_009664621
Phenuiviridae	*Goukovirus*	Cumuto virus	YP_009664615
Phenuiviridae	*Wenrivirus*	Wuhan horsefly Virus	YP_009305136
Phenuiviridae	*Tenuivirus*	Melon chlorotic spot virus	YP_009551587

Phenuiviridae	*Tenuivirus*	Rice stripe virus	NP_620522
Phenuiviridae	*Hudovirus*	Hubei lepidoptera virus 1	YP 009330283
Phenuiviridae	*Pidchovirus*	Pidgey bunyavirus M6	YP_009666272
Phenuiviridae	*Hudivirus*	Hubei diptera virus 4	YP_009330281
Phenuiviridae	*Beidivirus*	Hubei diptera virus 3	YP 009329894
Phenuiviridae	*Phasivirus*	Phasi Charoen-like virus	YP_009505332
Phenuiviridae	*Phasivirus*	Badu virus	YP_009505327
Phenuiviridae	*Horwuvirus*	Whenzhou Shrimp Virus 1	YP 009304989
Phenuiviridae	*Ixovirus*	Blacklegged tick phlebovirus 1	ANT80544
Phenuiviridae	*Uukuvirus*	Toyo virus	BCT55140.1
Phenuiviridae	*Uukuvirus*	Huangpi Tick Virus 2	YP 009293590
Phenuiviridae	*Uukuvirus*	Kabuto mountain virus	YP_009449450
Phenuiviridae	*Uukuvirus*	Precarious point virus	AEL29680
Phenuiviridae	*Uukuvirus*	Dabieshan Tick Virus	AJG39236
Phenuiviridae	*Uukuvirus*	Yongjia Tick Virus 1	AJG39274
Phenuiviridae	*Uukuvirus*	Okutama tick virus	BBK20268
Phenuiviridae	*Uukuvirus*	Changping Tick Virus 1	AJG39235
Phenuiviridae	*Uukuvirus*	Bole Tick Virus 1	AJG39234
Phenuiviridae	*Uukuvirus*	Brown dog tick phlebovirus 2	QDW81040
Phenuiviridae	*Uukuvirus*	Lihan tick virus	AJG39242
Phenuiviridae	*Uukuvirus*	Rhipicephalus associated phlebovirus 1	QCB64646
Phenuiviridae	*Phlebovirus*	Mukawa virus strain HJL	UUT07167
Phenuiviridae	*Phlebovirus*	Mukawa virus	YP00966332.1
Phenuiviridae	*Phlebovirus*	Kuriyama virus	BBF90225.1
Phenuiviridae	*Phlebovirus*	Sand fever Naples-like virus	AEL29673
Phenuiviridae	*Phlebovirus*	Cacao virus	QCI62731
Phenuiviridae	*Phlebovirus*	Adana virus	YP_009227127
Phenuiviridae	*Phlebovirus*	Rift Valley fever virus	YP_003848704
Phenuiviridae	*Phlebovirus*	Itaporanga virus	QCI62749
Phenuiviridae	*Phlebovirus*	Sandfly Sicilian Turkey virus	YP_004382742
Phenuiviridae	*Bandavirus*	Lone Star virus	YP_008003507
Phenuiviridae	*Bandavirus*	Bhanja virus	YP 009141013
Phenuiviridae	*Bandavirus*	Hunter Island virus	YP_008719916
Phenuiviridae	*Bandavirus*	Zwiesel bat banyangvirus	QHU78994
Phenuiviridae	*Bandavirus*	Heartland virus	YP 009047242
Phenuiviridae	*Bandavirus*	Guertu virus	ALQ33265
Phenuiviridae	*Bandavirus*	Phlebovirus XLL/China/2009	ADZ95575
Phenuiviridae	*Bandavirus*	FTLS virus	AHE38327
Phenuiviridae	*Bandavirus*	Orthobunyavirus BX-2010/Henan/CHN	AEO51773
Phenuiviridae	*Bandavirus*	Huaiyangshan virus	AFB82724
Phenuiviridae	*Bandavirus*	Severe fever with thrombocytopenia virus	QQZ00259
Nairoviridae	*Orthonairovirus*	Henan tick virus	QYW06749.1
Nairoviridae	*Orthonairovirus*	Huangpi Tick virus 1	AJG38237.1
Nairoviridae	*Orthonairovirus*	Tacheng Tick virus 1	YP 009304986
Nairoviridae	*Orthonairovirus*	Tamdy virus	QFU19352
Nairoviridae	*Orthonairovirus*	Vinegar Hill virus	AUD40046
Nairoviridae	*Orthonairovirus*	Abu Hammad virus	AMT75371
Nairoviridae	*Orthonairovirus*	Estero Real virus	AXP33563
Nairoviridae	*Orthonairovirus*	Soldado virus	AMT75425
Nairoviridae	*Orthonairovirus*	Raza virus	AMT75416
Nairoviridae	*Orthonairovirus*	Hughes orthonairovirus	AMT75407
Nairoviridae	*Orthonairovirus*	Keterrah virus	YP_009361838
Nairoviridae	*Orthonairovirus*	Gossas virus	ALD83626
Nairoviridae	*Orthonairovirus*	Qalyub orthonairovirus	AKC89319
Nairoviridae	*Orthonairovirus*	Leopards Hill virus	YP_009111284
Nairoviridae	*Orthonairovirus*	Yogue virus	YP 009246486
Nairoviridae	*Orthonairovirus*	Artashat virus	YP_009666119
Nairoviridae	*Orthonairovirus*	Taggert virus	QKK82908

Nairoviridae	*Orthonairovirus*	Tillamook virus	AMT75431
Nairoviridae	*Orthonairovirus*	Clo Mor virus	AMT75386
Nairoviridae	*Orthonairovirus*	Thiafora orthonairovirus	YP_009513191
Nairoviridae	*Orthonairovirus*	Erve virus	AMT75395
Nairoviridae	*Orthonairovirus*	Crimean Congo hemorrhagic fever virus	ASW20659
Nairoviridae	*Orthonairovirus*	Tofla virus	YP_009227122
Nairoviridae	*Orthonairovirus*	Nairobi sheep disease virus	AYI99257
Nairoviridae	*Striwavirus*	Sanxia Water Strider Virus 1	YP 009293594
Nairoviridae	*Shaspivirus*	Shayang Spider Virus 1	YP_009300680
Nairoviridae	*Unclassified*	South Bay virus	AII01810
Nairoviridae	*Unclassified*	Grotenhout virus	ARB16032
Nairoviridae	*Unclassified*	Norway nairovirus 1	ASY03236
Chuviridae	*Unclassified*	Wuhan Mosquito Virus 8	YP 009177719.1
Chuviridae	*Unclassified*	lmjin River virus 1	YP 009182177.1
Chuviridae	*Unclassified*	RNA-directed RNA Mos8Chu0 chuvirus	API61887.1
Chuviridae	*Unclassified*	Shuangao Fly Virus	AJG39080.1
Chuviridae	*Unclassified*	Hubei chuvirus-like virus1	YP 009337904.1
Chuviridae	*Unclassified*	Lishi spider virus 1	AJG39051.1
Chuviridae	*Unclassified*	Hubei chuvirus-like virus 3	YP 009337089.1
Chuviridae	*Unclassified*	Blacklegged tick chuvirus 2	AUW34382.1
Chuviridae	*Unclassified*	Wuchang Cockroach Virus 3	AJG39067.1
Chuviridae	*Unclassified*	Wenling chuvirus-like virus	YP 009333145.1
Chuviridae	*Unclassified*	Hubei odonate virus 11	YP 009336946.1
Chuviridae	*Unclassified*	Wuhan Louse Fly Virus 7	AJG39073.1
Chuviridae	*Unclassified*	Wuhan louse fly virus 6	AJG39070.1
Chuviridae	*Unclassified*	Changping Tick Virus 3	YP 009177707.1
Chuviridae	*Unclassified*	Tacheng Tick Virus 5	YP 009177717.1
Chuviridae	*Unclassified*	Wuhan tick virus 2	YP 009177722.1
Chuviridae	*Unclassified*	Lonestar tick chuvirus 1	YP 009254000.1
Chuviridae	*Unclassified*	Suffolk virus	YP 009177218.1
Chuviridae	*Unclassified*	Bole Tick Virus 3	YP 009177701.1
Chuviridae	*Unclassified*	Wenzhou crab virus 2	AJG39060.1
Chuviridae	*Unclassified*	Changping Tick Virus 2	YP 009177704.1
Chuviridae	*Unclassified*	Wenling chuvirus-like virus 2	YP 009333151.1
Chuviridae	*Unclassified*	Wenling crustacean virus 14	YP 009337856.1
Chuviridae	*Unclassified*	Wenling crustacean virus 13	YP 009337860.1
Chuviridae	*Unclassified*	Sanxia atyid shrimp virus 4	YP 009337428.1
Chuviridae	*Unclassified*	Tacheng Tick Virus 4	YP 009177716.1
Chuviridae	*Unclassified*	Beihai banacle virus 9	YP 009333178.1
Chuviridae	*Unclassified*	Hubei chuvirus-like virus 4	APG78769.1
Chuviridae	*Unclassified*	Xinzhou nematode virus 5	YP 009344984.1
Chuviridae	*Unclassified*	Wenling crustacean virus 15	YP 009336632.1
Chuviridae	*Unclassified*	Hubei myriapoda virus 8	YP 009330113.1
Chuviridae	*Unclassified*	Wenzhou Crab Virus 3	YP 009302836.1
Chuviridae	*Unclassified*	Beihal hermit crab virus 3	YP 009333157.1
Chuviridae	*Unclassified*	Shuangao Insect Virus 5	AJG39083.1
Chuviridae	*Unclassified*	Shayang Fly Virus 1	YP009300663.1
Chuviridae	*Unclassified*	Hubei coleoptera virus 3	YP 009336866.1
Chuviridae	*Unclassified*	Lyssavirus rabies	NP 056797.1

G protein			
**Family**	**Genus**	**Virus**	**Accession NO.**
Phenuiviridae	*Phlebovirus*	Mukawa virus	BBE15815.1
Phenuiviridae	*Phlebovirus*	Kuriyama virus	BBF90226.1
Phenuiviridae	*Phlebovirus*	Rift valley Fever virus	YP 003848705.1
Phenuiviridae	*Phlebovirus*	Toscana virus	YP_089671.1
Phenuiviridae	*Phlebovirus*	Sandfly fever Turkey virus	YP_004382742.1

Phenuiviridae	*Bandavirus*	SFTSV	YP_006504094.1
Phenuiviridae	*Uukuvirus*	Kabuto virus	YP 009449451.1
Phenuiviridae	*Uukuvirus*	Toyo virus	BCT55141.1
Phenuiviridae	*Uukuvirus*	Uukuniemi virus	NP_941979.1
Phenuiviridae	*Uukuvirus*	Kaisodi virus	YP 009551638.1
Phenuiviridae	*Uukuvirus*	Huangpi tick virus 2	YP_009293591.1
Phenuiviridae	*Uukuvirus*	Silverwater virus	YP_010086156.1

**N protein**			
**Family**	**Genus**	**Virus**	**Accession NO.**
Phenuiviridae	*Phlebovirus*	Mukawa virus	BBE15816.1
Phenuiviridae	*Phlebovirus*	Kuriyama virus	BBF90228.1
Phenuiviridae	*Phlebovirus*	Rift valley Fever virus	YP 003848707.1
Phenuiviridae	*Phlebovirus*	Toscana virus	YP_089668.1
Phenuiviridae	*Phlebovirus*	Sandfly fever Turkey virus	YP_004382744.1
Phenuiviridae	*Bandavirus*	SFTSV	YP 006504092.1
Phenuiviridae	*Uukuvirus*	Kabuto virus	YP_009449452.1
Phenuiviridae	*Uukuvirus*	Toyo virus	BCT55142.1
Phenuiviridae	*Uukuvirus*	Uukuniemi virus	NP 941980.1
Phenuiviridae	*Uukuvirus*	Kaisodi virus	YP_009551636.1
Phenuiviridae	*Uukuvirus*	Huangpi tick virus 2	YP_009293592.1
Phenuiviridae	*Uukuvirus*	Silverwater virus	YP 010086158.1

**Ns protein**			
**Family**	**Genus**	**Virus**	**Accession NO.**
Phenuiviridae	*Phlebovirus*	Mukawa virus	BBE15817.1
Phenuiviridae	*Phlebovirus*	Kuriyama virus	BBF90227.1
Phenuiviridae	*Phlebovirus*	Rift valley Fever virus	YP_003848706.1
Phenuiviridae	*Phlebovirus*	Toscana virus	YP_089667.1
Phenuiviridae	*Phlebovirus*	Sandfly fever Turkey virus	YP 004382745.1
Phenuiviridae	*Bandavirus*	SFTSV	YP_006504093.1
Phenuiviridae	*Uukuvirus*	Kabuto virus	YP_009449453.1
Phenuiviridae	*Uukuvirus*	Toyo virus	BCT55143.1
Phenuiviridae	*Uukuvirus*	Uukuniemi virus	NP_941981.1
Phenuiviridae	*Uukuvirus*	Kaisodi virus	YP_009551637.1
Phenuiviridae	*Uukuvirus*	Huangpi tick virus 2	YP 009293593.1
Phenuiviridae	*Uukuvirus*	Silverwater virus	YP_010086159.1

Supplementary Table 5 Nucleotide sequences used in this study

Family	Genus	Virus	Accession NO.		
			L	M	S
iFlaviridae	*iflavirus*	Haemaphysalis flava iflavirus	LC483655.1	\	\
iFlaviridae	*iflavirus*	Hubei tick virus 1	NC_032764.1	\	\
iFlaviridae	*iflavirus*	Hubei tick virus 3	NC_032751.1	\	\
iFlaviridae	*iflavirus*	Varroa destructor virus 1	NC_006494.1	\	\
iFlaviridae	*iflavirus*	Sacbrood virus	NC_002066.1	\	\
iFlaviridae	*iflavirus*	Infections flacherie virus	NC_003781.1	NC_043509.1	NC_043511.1
iFlaviridae	*iflavirus*	Perina nuda virus	NC_003113.1	LC133179.1	LC133180.1
Phenuiviridae	*Phlebovirus*	Mukawa virus	NC_043510.1	NC_014396.1	NC_014395.1
Phenuiviridae	*Phlebovirus*	Kuriyama virus	LC133178.1	NC_006320.1	NC_006318.1
Phenuiviridae	*Phlebovirus*	Rift valley Fever virus	NC_014397.1	NC_015411.1	NC_015413.1
Phenuiviridae	*Phlebovirus*	Toscana virus	NC_006319.1	NC_018138.1	NC_018137.1
Phenuiviridae	*Phlebovirus*	Sandfly fever Turkey virus	NC_015412.1	NC_036605.1	NC_036606.1
Phenuiviridae	*Bandavirus*	SFTSV	NC_018136.1	LC618932.1	LC618933.1
Phenuiviridae	*Uukuvirus*	Kabuto virus	NC_036604.1	NC_005220.1	NC_005221.1
Phenuiviridae	*Uukuvirus*	Toyo virus	LC618931.1	NC_040493.1	NC_040492.1
Phenuiviridae	*Uukuvirus*	Uukuniemi virus	NC_005214.1	NC_031139.1	NC_031140.1
Phenuiviridae	*Uukuvirus*	Kaisodi virus	NC_040494.1	NC_055369.1	NC_055371.1
Phenuiviridae	*Uukuvirus*	Huangpi tick virus 2	NC_031138.1	\	\
Phenuiviridae	*Uukuvirus*	Silverwater virus	NC_055370.1	\	\

Supplementary Figure 1

Amino acid alignment of the RdRp of Qingdao tick iflavirus, our HflFV and Japan HflFV

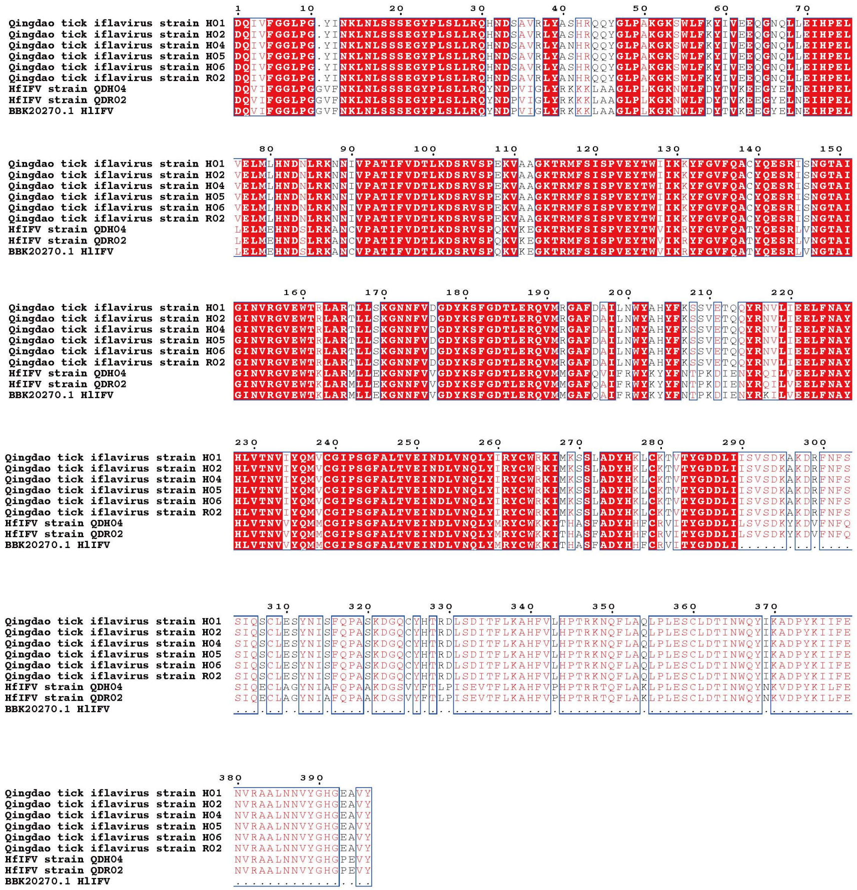

Supplementary Figure 2

Amino acid alignment of the RdRp of Qingdao tick phlebovirus , MKWV, MKWV strain HLJ and KURV

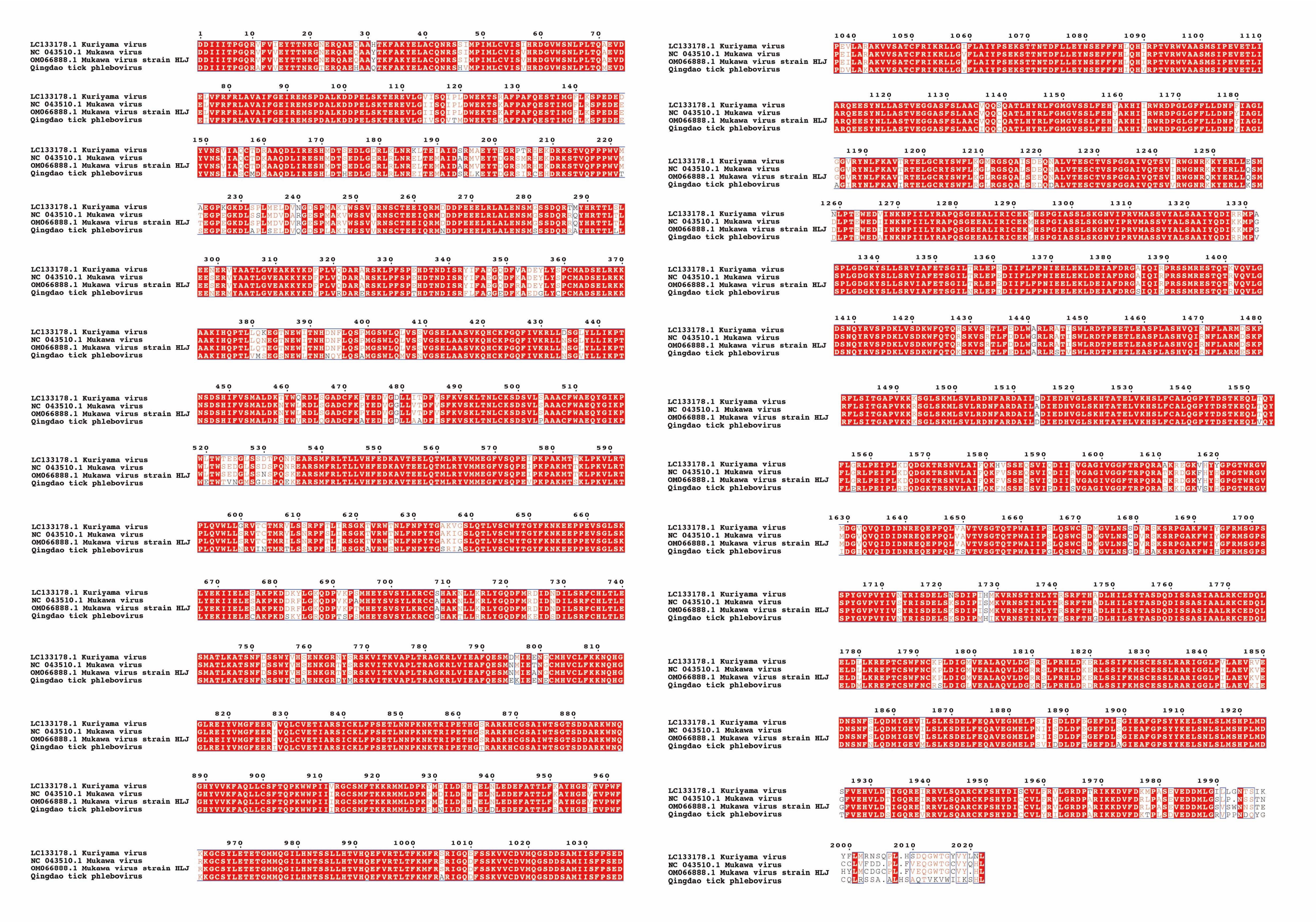

Supplementary Figure 3

Amino acid alignment of the glycoprotein precursor of Qingdao tick phlebovirus, MKWV, MKWV strain HLJ and KURV

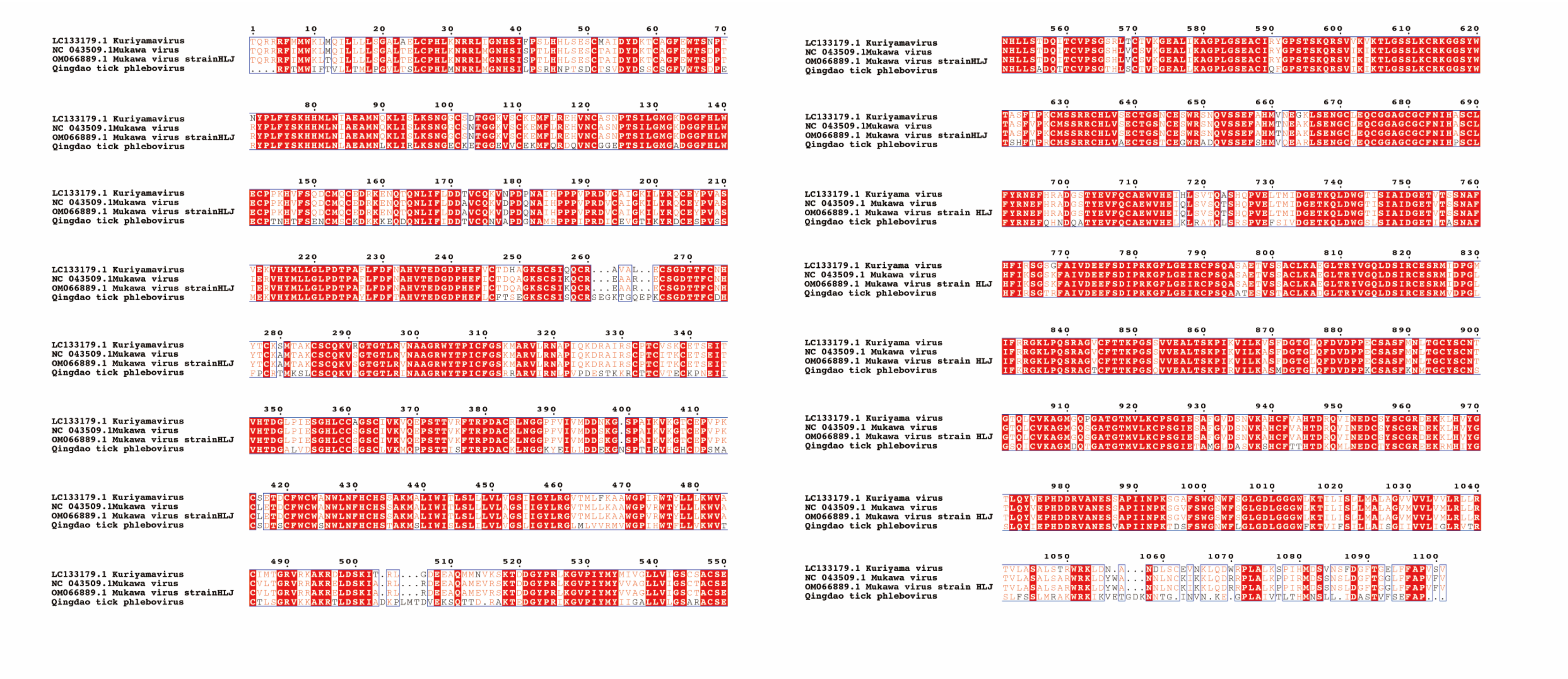

Supplementary Figure 4

Amino acid alignment of the N and Ns protein of Qingdao tick phlebovirus, MKWV, MKWV strain HLJ and KURV

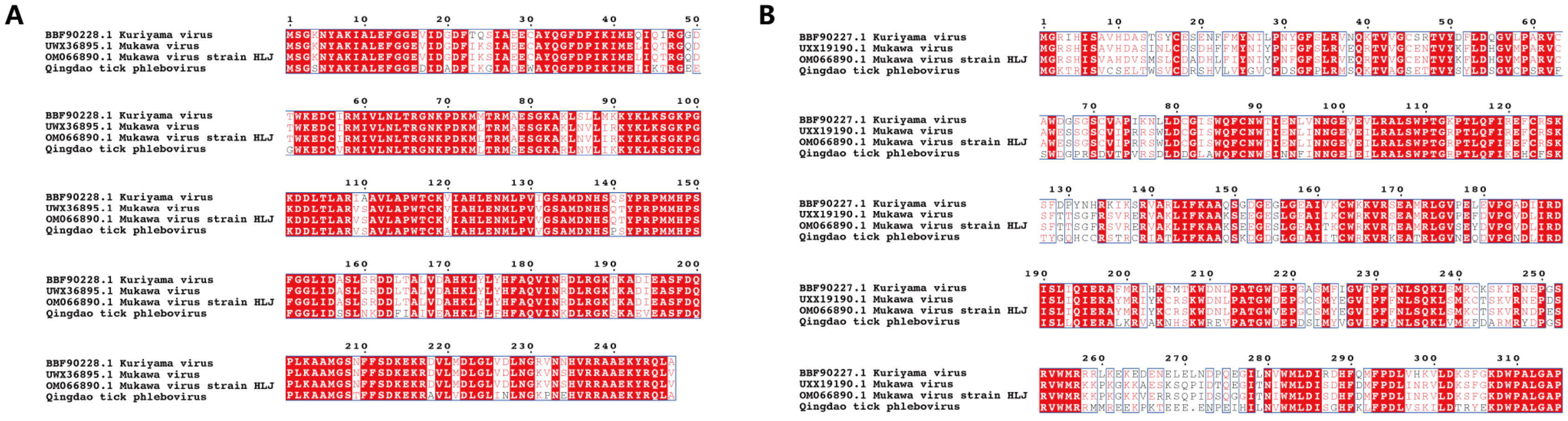

Supplementary Figure 5

Amino acid alignment of the RdRp of Qingdao tick uukuvirus and Toyo virus

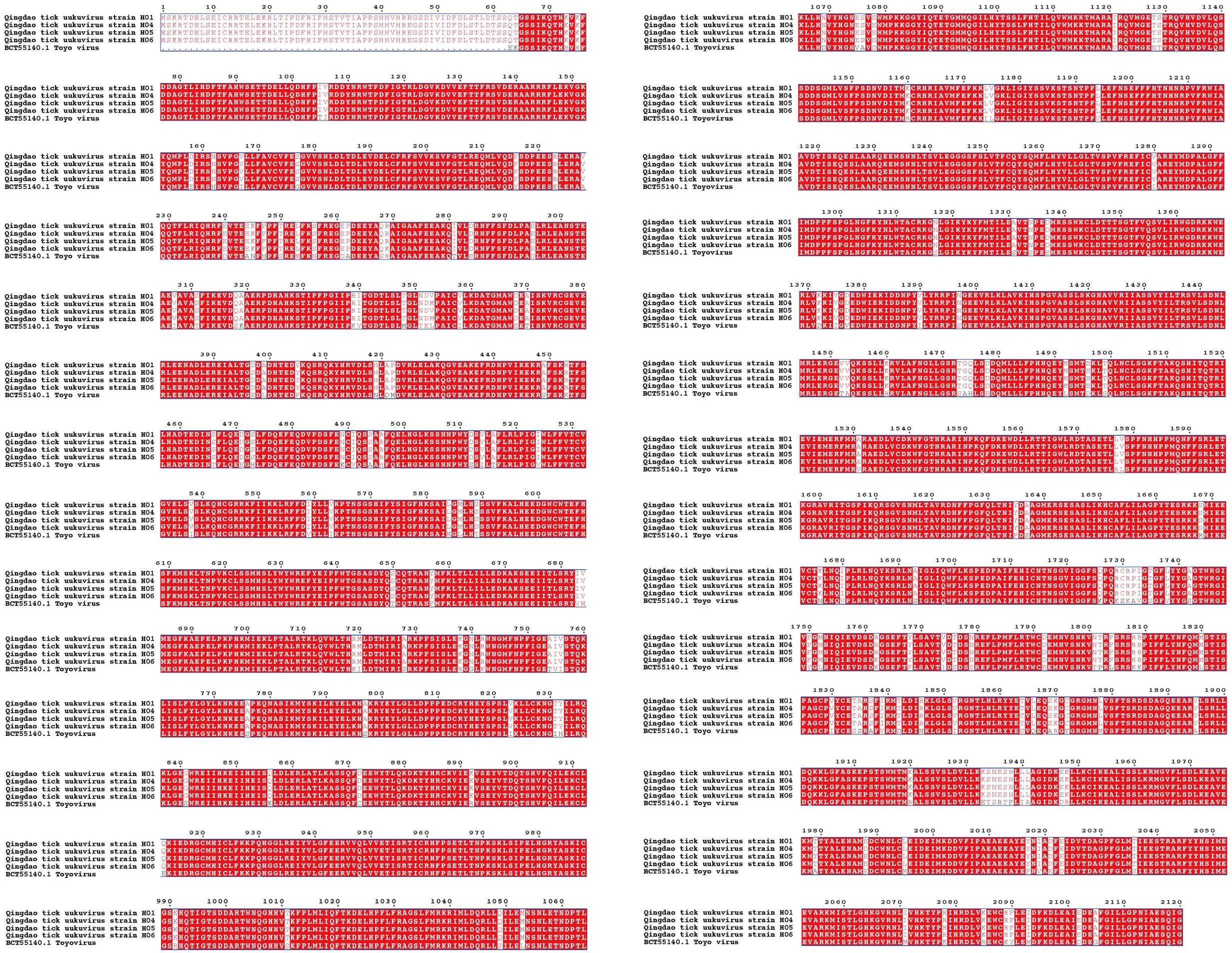

Supplementary Figure 6

Amino acid alignment of the glycoprotein precursor of Qingdao tick uukuvirus and Toyo virus

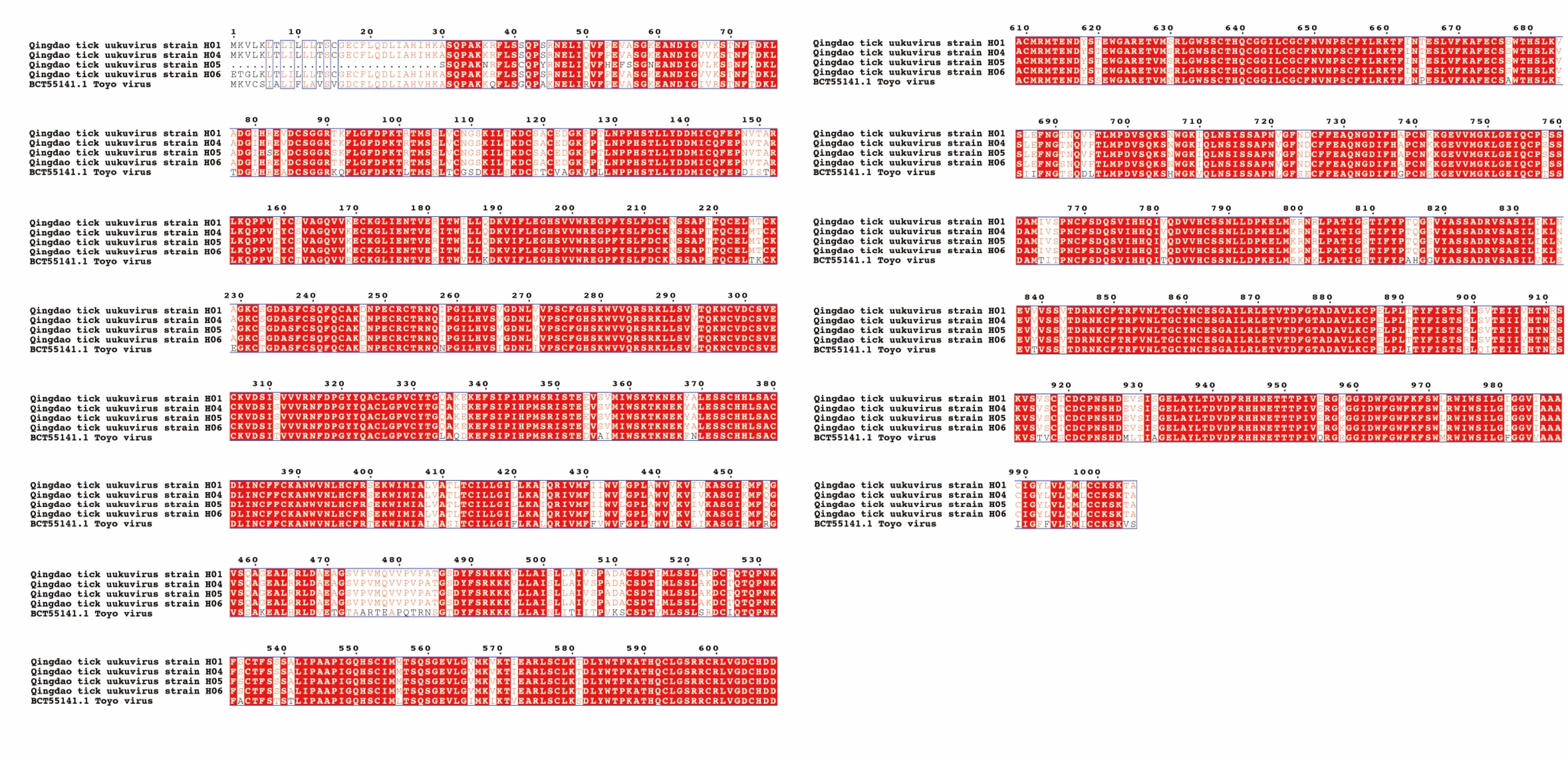

Supplementary Figure 7

Amino acid alignment of the the N and Ns protein of Qingdao tick uukuvirus, and Toyo virus

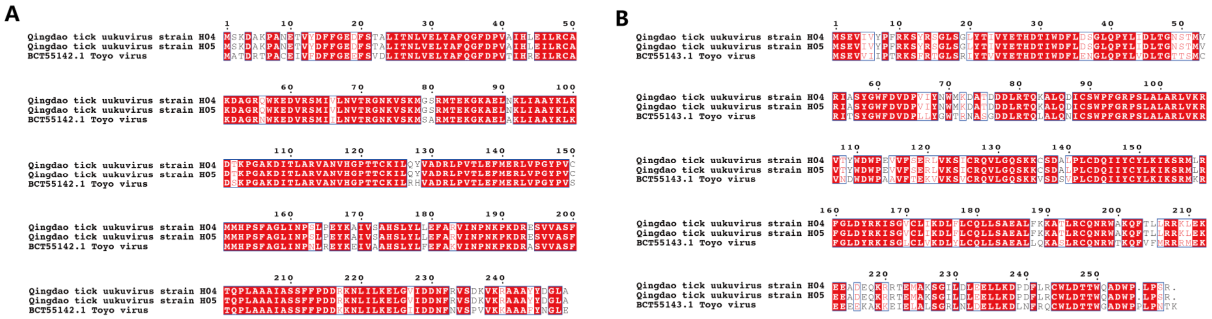

Supplement: Supplemental file 1 — Supplemental material. Download spectrum.05340-22-s0001.pdf, PDF file, 7.7 MB [file spectrum.05340-22-s0001.pdf]
